# Adaptive Strategy of the Perennial Halophyte Grass *Puccinellia tenuiflora* to Long-Term Salinity Stress

**DOI:** 10.3390/plants13233445

**Published:** 2024-12-08

**Authors:** Lei Han, Zhanwu Gao, Luhao Li, Changyou Li, Houxing Yan, Binbin Xiao, Yimeng Ma, Huan Wang, Chunwu Yang, Hongwei Xun

**Affiliations:** 1 Key Laboratory of Molecular Epigenetics of Ministry of Education, Northeast Normal University, Changchun 130024, China; hanl335@nenu.edu.cn (L.H.); lilh860@nenu.edu.cn (L.L.); hxyan@nenu.edu.cn (H.Y.); xiaobb663@nenu.edu.cn (B.X.); maym057@nenu.edu.cn (Y.M.); 2 Tourism and Geographical Science Institute, Baicheng Normal University, Baicheng 137000, China; gzw@bcnu.edu.cn; 3 School of Life Science, Jilin Normal University, Siping 136000, China; licy858@163.com; 4 Department of Agronomy, Jilin Agricultural University, Changchun 130018, China; angelfuture@163.com

**Keywords:** *Puccinellia tenuiflora*, salinity stress, metabolites, plant hormones

## Abstract

Salinity stress influences plants throughout their entire life cycle. However, little is known about the response of plants to long-term salinity stress (LSS). In this study, *Puccinellia tenuiflora*, a perennial halophyte grass, was exposed to 300 mM NaCl for two years (completely randomized experiment design with three biological replicates). We measured the photosynthetic parameters and plant hormones and employed a widely targeted metabolomics approach to quantify metabolites. Our results revealed that LSS induced significant metabolic changes in *P. tenuiflora*, inhibiting the accumulation of 11 organic acids in the leaves and 24 organic acids in the roots and enhancing the accumulation of 15 flavonoids in the leaves and 11 phenolamides in the roots. The elevated accumulation of the flavonoids and phenolamides increased the ability of *P. tenuiflora* to scavenge reactive oxygen species. A comparative analysis with short-term salinity stress revealed that the specific responses to long-term salinity stress (LSS) included enhanced flavonoid accumulation and reduced amino acid accumulation, which contributed to the adaptation of *P. tenuiflora* to LSS. LSS upregulated the levels of abscisic acid in the leaves and ACC (a direct precursor of ethylene) in the roots, while it downregulated the levels of cytokinins and jasmonic acids in both the organs. These tolerance-associated changes in plant hormones would be expected to reprogram the energy allocation among growth, pathogen defense, and salinity stress response. We propose that abscisic acid, ethylene, cytokinins, and jasmonic acids may interact with each other to construct a salinity stress response network during the adaptation of *P. tenuiflora* to LSS, which mediates salinity stress response and significant metabolic changes. Our results provided novel insights into the plant hormone-regulated metabolic response of the plants under LSS, which can enhance our understanding of plant salinity tolerance.

## 1. Introduction

Soil salinization is a significant threat to agricultural production and food security in arid and semi-arid areas [1]. Halophytes are remarkable plants that can survive under high salinity conditions that would kill 99% of other plant species; some extreme halophytes survive even under 1200 mM NaCl [2]. Although halophytes and glycophytes displayed dramatic differences in salinity tolerance levels, they share most of the salinity tolerance mechanisms, including the control of Na^+^ in roots, compartmentalization of Na^+^ among organs or within cells, and synthesis of compatible solutes in the cytoplasm [3,4]. The selection pressure of extreme salinity conditions has driven halophytes to evolve unique structural (e.g., salt gland) and metabolic characteristics to adapt to high salinity environments [5]. Halophytes achieve strong salinity tolerance through enhancing activities of ion exchanger/transporter [6,7] and antioxidant enzymes [8], accumulating higher concentrations of compatible solutes [9,10], and synthesizing more non-enzymatic antioxidants [11,12].

Multiple lines of evidence indicate that halophytes can mediate their salinity tolerance response through the regulation of plant hormones, including abscisic acid (ABA), ethylene (ETH), jasmonic acid (JA), and cytokinins (CKs) [4,9,13]. ABA coordinates with the second messenger, Ca^2+^, to help plants cope with osmotic stress through stomatal closure under salinity stress [14,15,16]. Similarly, ETH also plays a positive role in salinity stress tolerance by enhancing nitrate assimilation and maintaining Na^+^/K^+^ homeostasis [17]. There is evidence suggesting an antagonistic relationship between CKs and ABA/ETH. Exogenous application of CKs can downregulate ABA-mediated stress response genes and ethylene response factor genes (*ERF1* and *ERF5*) [18].

In saline grassland, salinity stress influences the living plants for their entire life cycle. The effects of short-term salinity stress (SSS) and long-term salinity stress (LSS) on plants are different. SSS damages plant growth through osmotic stress and ion toxicity [19], while LSS may lead to excessive accumulation of toxic ions and nutritional and metabolic imbalances. Understanding the plant response to LSS is helpful for improving the salinity tolerance of plants, especially perennial forage grass. However, the response of plants to LSS was unclear. The perennial herbaceous halophytic grass *Puccinellia tenuiflora* is a dominant species in the saline grasslands of the Songnen Plain in Northeast China, with a high forage value and remarkable salinity tolerance, since it is able to thrive under extreme soil salinity conditions (2–3% soil salinity) [20]. Therefore, many researchers have explored the genetic and physiological mechanisms by which this species resists SSS, including gene function [21,22,23], ion homeostasis [24,25], photosynthetic characteristics [26], gene expression [3,27,28], proteomics [29], and metabolomics [30,31].

It has been reported that *P. tenuiflora* maintains a low Na^+^ level via limiting Na^+^ influx into roots, which results in a higher selectivity for K^+^ over Na^+^ under salinity stress [32]. *PutHKT1;5* and *PutSOS1* may play an important role in ion balance by mediating Na^+^ retrieval from shoots to roots in *P. tenuiflora* [33]. Our previous research showed the expansion of several K^+^ uptake gene families and flavonoid biosynthesis gene families during the evolution of *P. tenuiflora*, which contributes to the formation of its salinity tolerance [3]. The accumulation of flavonoids is an important adaptive response of *P. tenuiflora* to high salinity conditions [3,34,35]. Extensive secretion of phenolic acids and fatty acids promotes rhizosphere pH regulation and contributes to the alkali tolerance of *P. tenuiflora* [35]. Overexpression of the *P. tenuiflora* rubredoxin family protein gene (*PutRUB*) and *PutNHX* improved the alkali stress tolerance of transgenic *Arabidopsis thaliana* plants through decreasing H_2_O_2_ accumulation [36,37]. Overexpression of *PutAMT1;1* promoted early root growth after seed germination in transgenic *A. thaliana* plants under salinity stress via alleviating ammonia toxicity [38]. Although an increasing number of researchers have explored the SSS tolerance of *P. tenuiflora*, its LSS tolerance mechanism is unclear. To fill this knowledge gap, we conducted a comprehensive study of the photosynthetic parameters, metabolic profiling, and plant hormone response in *P. tenuiflora* after two years of exposure to LSS.

## 2. Results

### 2.1. Photosynthesis

**Figure 1:**
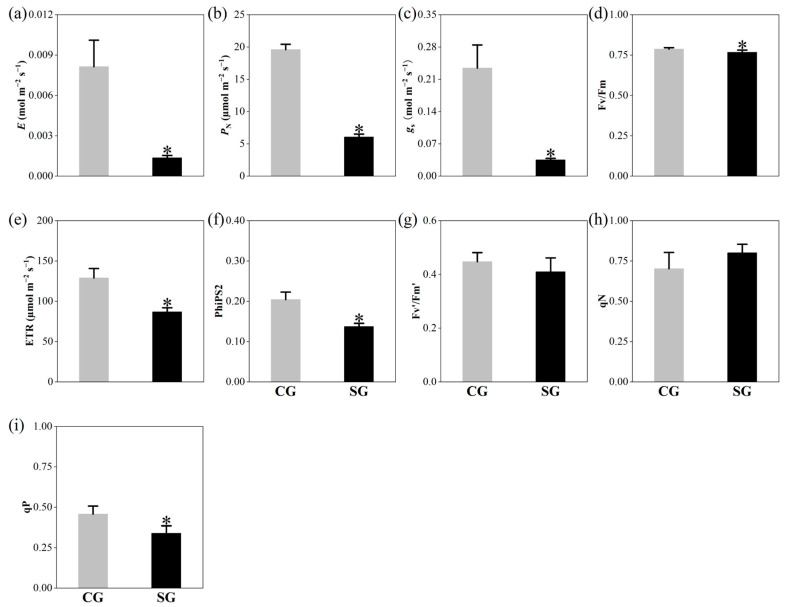
Effects of long-term salinity stress on photosynthesis of *Puccinellia tenuiflora*. (**a**) *E*, transpiration rate; (**b**) *P*_N_, net photosynthetic rate; (**c**) *gs*, stomatal conductance; (**d**) Fv/Fm, maximum quantum efficiency of photosystem II (PSII); (**e**) ETR, electron transport rate; (**f**) PhiPS2, real quantum efficiency of PSII; (**g**) Fv′/Fm′, effective quantum efficiency of PSII; (**h**) qN, non-photochemical quenching; (**i**) qP, photochemical quenching. *P. tenuiflora* was treated with a nutrient solution with 300 mM NaCl (stress treatment group, SG) or without NaCl (control group, CG) for two years. Each treatment has three biological replicates. The values are expressed as means of three biological replicates (±S.D.). The asterisk indicates a significant difference between control and stress treatments (*t*-test, *p* < 0.05).

LSS strongly inhibited the photosynthesis of *P. tenuiflora*, resulting in a significant decrease in net photosynthetic rate (*P*_N_), stomatal conductance (*g_s_*), transpiration rate (*E*), electron transport rate (ETR), real quantum efficiency of PSII (PhiPS2), and photochemical quenching (qP) (Figure 1 and Table S1). 

### 2.2. Membrane Damage

**Figure 2:**
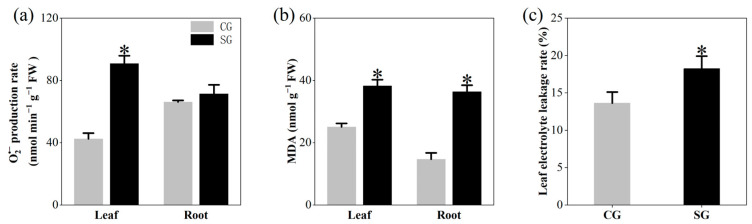
Effects of long-term salinity stress on membrane damage of *Puccinellia tenuiflora*. (**a**) superoxide (O_2_^•−^) production rate; (**b**) malondialdehyde (MDA) concentration; (**c**) leaf electrolyte leakage rate. *P. tenuiflora* was supplied with a nutrient solution with 300 mM NaCl (stress treatment group, SG) or without NaCl (control group, CG) for two years. Each treatment has three biological replicates. The values are expressed as means of three biological replicates (±S.D.). The asterisk indicates a significant difference between control and stress treatments (*t*-test, *p* < 0.05).

LSS increased the leaf O_2_^•−^ concentration by 114.17% but had no significant effect on the roots relative to the control plants (Figure 2a). Additionally, LSS significantly increased the concentration of malondialdehyde (MDA) in both the leaves and roots, with the increase being more pronounced in roots (147.27%) than in leaves (52.73%) (Figure 2b and Table S2). The results also showed that LSS increased the leaf electrolyte leakage rate by 33.69% (Figure 2c and Table S2).

### 2.3. Metabolic Response 

#### 2.3.1. Metabolic Profile

**Figure 3:**
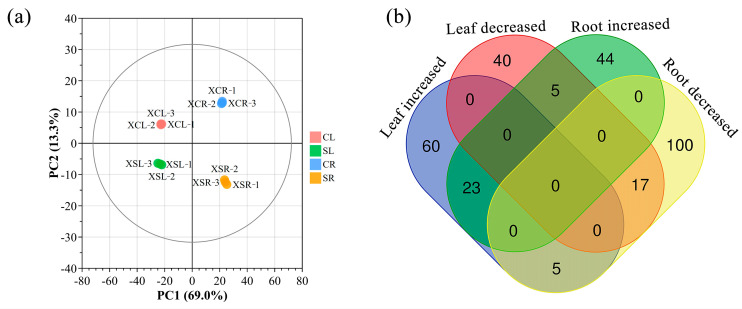
The principal component analysis of the metabolic profiles (**a**) and the Venn diagram of differentially accumulated metabolites (**b**) in *Puccinellia tenuiflora. P. tenuiflora* was supplied with a nutrient solution containing (stress treatment) or not (control) 300 mM NaCl for two years. Each treatment has three biological replicates.

To gain deeper insights into the metabolic response of *P. tenuiflora* to LSS, we employed the widely targeted metabolomic approach to detect metabolites in the leaves and roots of plants subjected to LSS. In total, we identified 835 metabolites, including 87 amino acids or amino acid derivatives, 72 organic acids, 50 carbohydrates, 15 vitamins, 132 lipids, 60 alkaloids, 177 flavonoids, 14 lignans, 11 coumarins, 54 nucleotides or nucleotide derivatives, 139 phenolic acids, 11 terpenoids, six tannins, one quinone, and six other metabolites. LSS increased the concentrations of 88 metabolites in the leaves and 72 metabolites in the roots. LSS also significantly reduced the concentrations of 62 metabolites in the leaves and 122 metabolites in the roots. The Venn diagram in Figure 3b visually displays the number of differentially accumulated metabolites under control and LSS conditions (|Log2 fold change (stress/control)| ≥ 1, VIP > 1, *p* < 0.05). We found that the concentrations of 23 metabolites were significantly enhanced in both the roots and leaves, whereas 65 metabolites were upregulated in the leaves but not in the roots, and 49 metabolites were upregulated in the roots but not in the leaves. The concentrations of only 17 metabolites were lowered in both the roots and leaves (Figure 3b).

**Figure 4:**
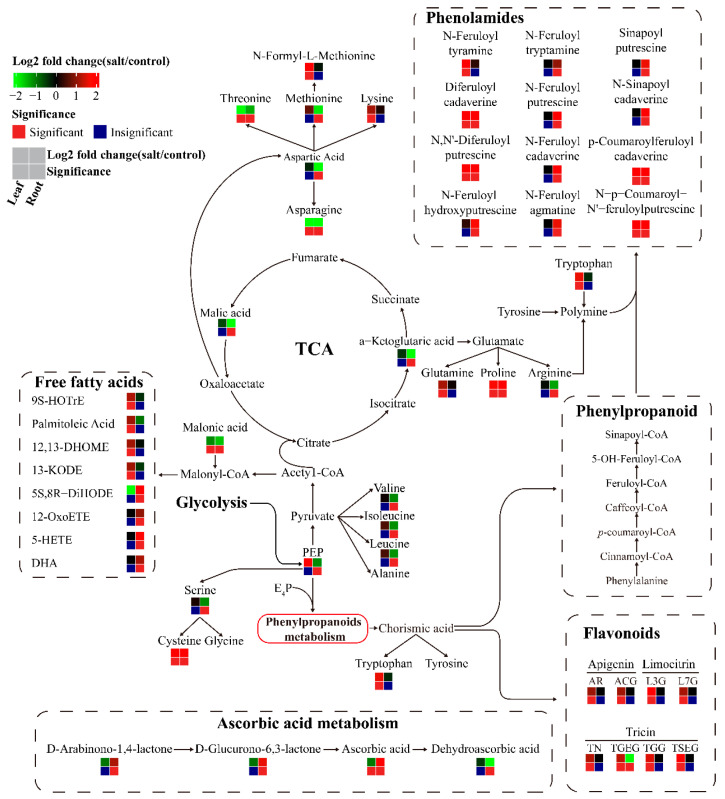
Metabolic response of *Puccinellia tenuiflora* to long-term salinity stress. *P. tenuiflora* was supplied with a nutrient solution with 300 mM NaCl (stress treatment) or without NaCl (control) for two years. Each treatment has three biological replicates. The red color indicates a significant difference between control and stress treatments. TCA, tricarboxylic acid; PEP, phosphoenolpyruvate; E_4_P, erythritose-4-phosphate; AR, apigenin-7-O-rutinoside; ACG, apigenin-7-O-(6″-p-coumaryl)glucoside; L3G, limocitrin-3-O-glucoside; L7G, limocitrin-7-O-glucoside; TN, tricin-7-O-neohesperidoside; TSEG, tricin-*4*′*-O*-(syringyl alcohol)ether-*5-O*-glucoside; TGG, Tricin-4′-O-glucoside-7-O-glucoside; TGEG, tricin-*4*′*-O*-(guaiacylglycerol)ether-*7-O*-glucoside; 13-KODE, (*9Z*,*11E*)-13-Oxooctadeca-9,11-dienoic acid; 12,13-DHOME, (*9Z*)-12,13-dhydroxyoctadec-9-enoic acid; 5S,8R-DiHODE, (*5S*,*8R*,*9Z*,*12Z*)-5,8-dihydroxyoctadeca-9,12-dienoate; 9S-HOTrE, 9-hydroxy-10,12,15-octadecatrienoic acid; 12-OxoETE, 12-oxo-5,8,10,14-eicosatetraenoic acid; 5-HETE, 5-hydroxy-6,8,11,14-eicosatetraenoic acid; DHA, cis-4,7,10,13,16,19-docosahexaenoic acid.

To determine whether the metabolomic signature of stressed *P. tenuiflora* plants was different from that of the control plants, we conducted PCA analysis for metabolic profiling. The results showed that the first principal component (PC1) distinguished the leaves and roots and explained 69.0% of the total variation (Figure 3a). The second principal component (PC2) significantly separated the control and stress treatments and explained 13.3% of the total variation. The three replicates of each treatment or organ were close or coincident, indicating that there was a close agreement among the replicates (Figure 3a). The metabolic pathway map showed that salinity stress enhanced the accumulation of amino acids, amino acid derivatives, free fatty acids, carbohydrates, phenolamides, flavonoids, and ascorbic acid (AsA) but decreased the concentrations of organic acids, such as malic acid (Figure 4).

#### 2.3.2. Organic Osmotic Solutes

**Figure 5:**
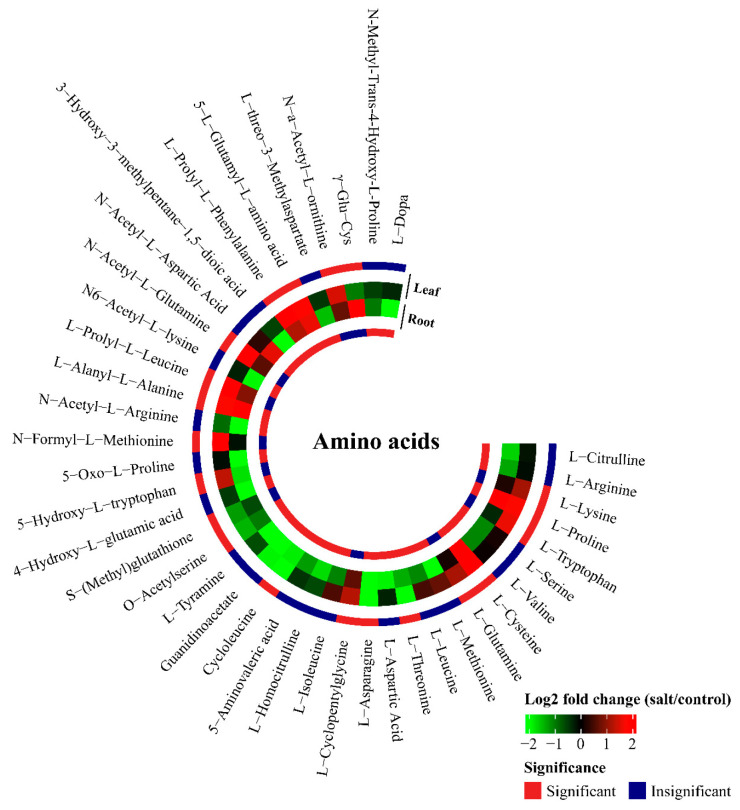
Effects of long-term salinity stress on the relative concentrations of amino acids and amino acid derivatives in *Puccinellia tenuiflora. P. tenuiflora* was irrigated with a nutrient solution containing (stress treatment) or not (control) 300 mM NaCl for two years. Each treatment has three biological replicates. The red color indicates a significant difference between control and stress treatments. L-Dopa, 3,4-dihydroxy-L-phenylalanine.

**Figure 6:**
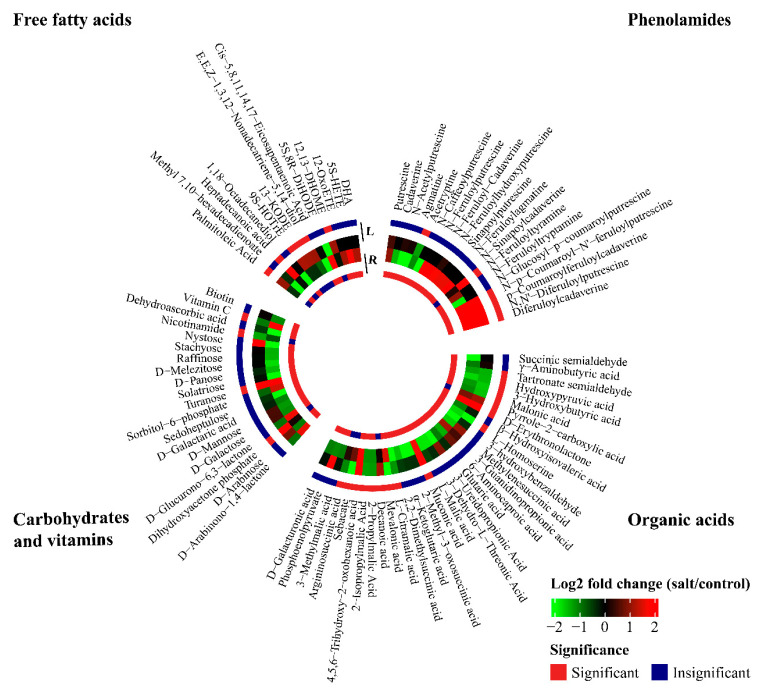
Effects of long-term salinity stress on the relative concentrations of organic acids, phenolamides, free fatty acids, carbohydrates, and vitamins in *Puccinellia tenuiflora. P. tenuiflora* was irrigated with a nutrient solution containing (stress treatment) or not (control) 300 mM NaCl for two years. The red color indicates a significant difference between control and stress treatments. 13-KODE, (*9Z*,*11E*)-13-Oxooctadeca-9,11-dienoic acid; 12,13-DHOME, (*9Z*)-12,13-dihydroxyoctadec-9-enoic acid; 5S,8R-DiHODE, (5S,8R,9Z,12Z)-5,8-dihydroxyoctadeca-9,12-dienoate; 9S-HOTrE, 9-hydroxy-10,12,15-octadecatrienoic acid; 12-OxoETE, 12-oxo-5,8,10,14-eicosatetraenoic acid; 5S-HETE, 5-hydroxy-6,8,11,14-eicosatetraenoic acid; DHA, cis-4,7,10,13,16,19-docosahexaenoic acid.

LSS significantly changed the concentrations of 103 organic osmotic solutes, namely 41 amino acids or amino acid derivatives, 33 organic acids, 16 carbohydrates, 4 vitamins, and 13 free fatty acids (Table S3). The concentrations of 14 amino acids or amino acid derivatives in the leaves were increased under LSS (Figure 5). Of these upregulated amino acids or amino acid derivatives, the concentrations of N-acetyl-L-glutamine, L-prolyl-L-leucine, and L-prolyl-L-phenylalanine in the leaves were enhanced by 5.8-, 5.4-, and 5.4-folds under LSS, respectively. LSS reduced the concentrations of six amino acids or amino acid derivatives in the leaves, namely γ-Glu-Cys (0.49-fold), *S*-(methyl) glutathione (0.41-fold), *O*-acetylserine (0.36-fold), L-threonine (0.23-fold), L-asparagine (0.00009-fold), and cycloleucine (0.00001-fold) (Figure 5). However, in the roots, the concentrations of only six amino acids or amino acid derivatives were elevated under LSS, whereas the concentrations of 24 amino acids or amino acid derivatives were significantly lowered (Figure 5). In response to LSS, the concentrations of many organic acids were reduced in both the leaves and roots (Figure 6). The concentrations of 11 organic acids significantly decreased in the leaves of the stressed plants, whereas those of the 24 organic acids significantly decreased in the roots (Figure 6). Many of the organic acids showing decreased accumulation were “hub” metabolites. For instance, malonic acid (0.34-fold in the leaves and 0.45-fold in the roots) is an important substrate for lipid metabolism; L-citramalic acid (0.43-fold in leaves and 0.00005-fold in roots), 2-propylmalic acid (0.41-fold in the leaves and 0.43-fold in the roots), and 2-isopropylmalic acid (0.40-fold in the leaves and 0.43-fold in the roots) are involved in pyruvate metabolism (Figure 6). LSS enhanced the accumulation of two carbohydrates in the leaves and four carbohydrates in the roots (Figure 6), but it decreased the concentrations of 11 carbohydrates in the roots (Figure 6). The concentrations of many free fatty acids in the leaves were greatly changed by LSS. The concentrations of six free fatty acids in the leaves were significantly increased after LSS (Figure 6). Of these fatty acids, the fold increase in heptadecanoic acid reached 5681.26 (Figure 6). However, in the roots, the concentrations of four fatty acids increased, while those of three fatty acids decreased after LSS (Figure 6). 

#### 2.3.3. Non-Enzymatic Antioxidants

**Figure 7:**
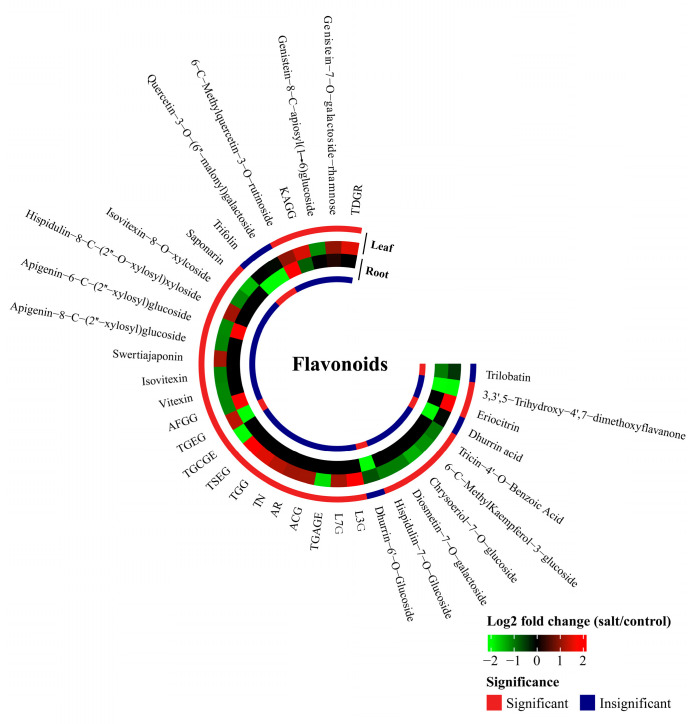
Effects of long-term salinity stress on the relative concentrations of flavonoids in *Puccinellia tenuiflora. P. tenuiflora* was irrigated with a nutrient solution containing (stress treatment) or not (control) 300 mM NaCl for two years. Each treatment has three biological replicates. The red color indicates a significant difference between control and stress treatments. TGAGE, tricin-4′-*O*-[β-guaiacyl-(9″-*O*-acetyl)glycerol]ether; TSEG, tricin-4′-*O*-(syringyl alcohol)ether-5-*O*-glucoside; TGCGE, tricin-4′-*O*-[β-guaiacyl-(9″-*O*-p-coumaroyl)glycerol]ether; TGEG, tricin-4′-*O*-(guaiacylglycerol)ether-7-*O*-glucoside; AFGG, apigenin-7-*O*-(2″-feruloyl)glucuronide-4′-*O*-glucuronide; KAGG, kaempferol-3-*O*-(6″-acetyl)glucosyl-(1→3)-galactoside; TDGR, 5,7,4′-trihydroxy-6,8-dimethoxyisoflavone-7-*O*-galactoside-rhamnose.

After LSS, the concentrations of 36 flavonoids (18 flavonoids, four flavonols, three isoflavones, eight flavonoid carbonosides, two dihydroflavones, and one chalcone) were significantly changed in the roots or leaves (Figure 7 and Table S3). LSS increased the concentrations of 15 flavonoids in the leaves, although the concentrations of none of the flavonoids were significantly increased in the roots (Figure 7). LSS decreased the concentrations of 16 flavonoids in the leaves and six flavonoids in the roots (Figure 7). LSS increased the concentrations of five phenolamides in the leaves and 12 phenolamides in the roots, while it decreased one phenolamide in the leaves and six phenolamides in the roots (Figure 6). The concentrations of *p*-coumaroylferuloylcadaverine, *N*-*p*-coumaroyl-*N′*-feruloylputrescine, and diferuloylcadaverine were increased by 53.73-, 47.13-, and 12.45-folds in the leaves, respectively. In the roots, the levels of *p*-coumaroylferuloylcadaverine, *N*-feruloylputrescine, and *N*-*p*-coumaroyl-*N′*-feruloylputrescine were enhanced under LSS by 8.99-, 5.86- and 5.79-folds, respectively. Four vitamins (nicotinamide, dehydroascorbic acid, AsA, and biotin) were differentially accumulated under control and stress conditions (Figure 6). After LSS treatment, the concentrations of dehydroascorbic acid and biotin were reduced in the roots, whereas the level of AsA was enhanced in the roots but decreased in the leaves (Figure 6).

### 2.4. Plant Hormones

**Figure 8:**
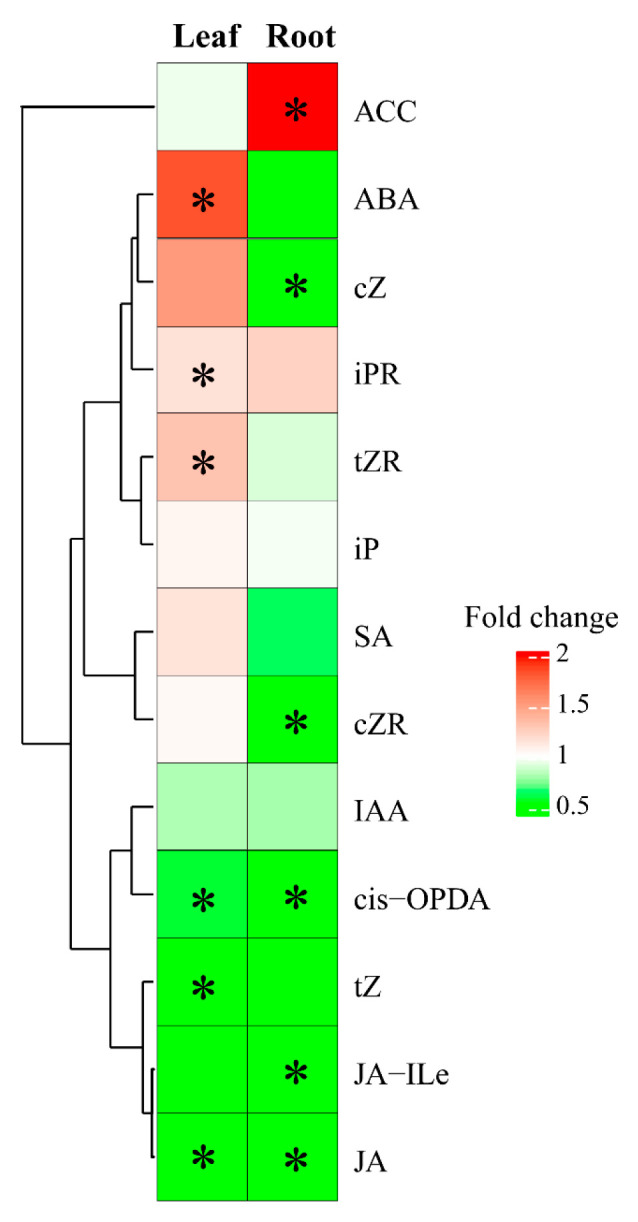
Effects of long-term salinity stress on the concentrations of plant hormones in *Puccinellia tenuiflora. P. tenuiflora* was irrigated with a nutrient solution containing (stress treatment) or not (control) 300 mM NaCl for two years. Each treatment has three biological replicates. The asterisk indicates a significant difference between control and stress treatments (*t*-test, *p* < 0.05).

In this study, we determined the concentrations of 21 plant hormones, namely four gibberellins (GA_1_, GA_3_, GA_4_, and GA_7_), three jasmonic acids (JAs) (JA, jasmonic acid-isoleucine (JA-ILe), and *cis*-OPDA), ABA, the auxin indole-3-acetic acid (IAA), salicylic acid (SA), seven CKs (dihydrozeatin, *trans*-zeatin (tZ), *cis*-zeatin (cZ), isopentenyladenosine (iPR), isopentenyladenine (iP), *trans*-zeatin riboside (tZR), and *cis*-zeatin riboside (cZR)), 1-aminocyclopropane-1-carboxylic acid (ACC, a direct precursor of ethylene), and three brassinosteroids (oleuropein lactone, chelizidone, and champaziol). A total of 13 plant hormones were detected in the roots or leaves, namely six CKs, three JAs, IAA, ABA, SA, and ACC (Figure 8 and Table S4). Of the 13 plant hormones detected, the concentrations of ten plant hormones were significantly affected by LSS, namely ABA ACC, three JAs (*cis*-OPDA, JA, and JA-ILe), and five CKs (tZ, tZR, cZ, cZR, and iPR) (Figure 8). Exposure to LSS increased the levels of ABA and two CKs (tZR and iPR) in the leaves and ACC in the roots (Figure 8). However, LSS decreased the concentrations of JA, *cis*-OPDA, and tZ in the leaves, and it lowered the levels of two CKs (cZ and cZR) and three JAs (*cis*-OPDA, JA, and JA-ILe) in the roots (Figure 8).

## 3. Discussion

Salinity stress causes negative effects on plants via osmotic stress, ion toxicity, nutritional disorders, and oxidative stress due to ROS damage [4,19,39]. In this study, we observed that LSS enhanced the concentrations of ROS and MDA (the product of lipid peroxidation caused by excess ROS) in both the leaves and roots of *P. tenuiflora* (Figure 2), indicating severe damage due to excessive ROS accumulation. The plants generally change their metabolism to cope with various damages caused by salinity stress [40]. In the present study, LSS inhibited the accumulation of organic acids in *P. tenuiflora*, including even “hub” metabolites of the TCA pathway, such as malic acid and α-ketoglutaric acid (Figure 4 and Figure 6). However, LSS promoted the accumulation of ROS scavengers, including 20 phenylpropanoid metabolites (five phenolamides and 15 flavonoids), as well as 14 amino acids or amino acid derivatives in the leaves, 12 phenolamides, six amino acids or amino acid derivatives, and ascorbic acid in the roots of *P. tenuiflora* (Figure 4, Figure 5, Figure 6 and Figure 7). Our previous study demonstrated that LSS treatment could induce hypomethylation of the promoter region of many genes involved in flavonoids and phenolamides biosynthesis in *P. tenuiflora* [41]. However, when *P. tenuiflora* was exposed to SSS, salinity stress did not upregulate the expression of genes involved in the synthesis of flavonoids and phenolamides [3]. Additionally, LSS enhanced the accumulation of cysteine, glutamine, lysine, proline, and tryptophan in the leaves of *P. tenuiflora* (Figure 5), whereas the enhanced accumulations were not observed in the short-term stressed *P. tenuiflora* leaves [41]. LSS did not affect the accumulation of fructose, sucrose, and maltose in the leaves of *P. tenuiflora* (Figure 6), but the levels of the three carbohydrates were reduced in the short-term stressed *P. tenuiflora* leaves [41]. The above data revealed that *P. tenuiflora* employed different mechanisms to cope with LSS and SSS. Flavonoids, reducing carbohydrates, and reducing amino acids all consist of the active groups to scavenge ROS, which contributed to the adaptation of *P. tenuiflora* to LSS.

Flavonoids and phenolamides from phenylpropanoid metabolites have been identified to play important roles in ROS scavenging under salinity stress [42]. Kiani et al. (2021) reported that salinity stress significantly increased the levels of apigenin, luteolin, and rutin in wheat and *Aegilops cylindrica* [43]. In the present study, the concentrations of 15 flavonoids were upregulated in the leaves but not in the roots (Figure 4 and Figure 7). The upregulated flavonoids were all glycosides, while their aglycone C_6_-C_3_-C_6_ skeletons were mainly apigenin, limocitrin, and tricin (Figure 4 and Figure 7). Due to the presence of 2,3-unsaturated double bonds and a 4-carbonyl group, these compounds have strong antioxidant abilities [44]. Glycosides increase the water solubility of the flavonoid aglycone and facilitate their long-distance transport or intracellular transport to organelles, thereby effectively scavenging ROS in specialized locations of *P. tenuiflora* [45]. Phenolamides are derived from the combination of hydroxycinnamic acid derivatives and aliphatic or aromatic amines [46]. The important roles of phenolamides in plant defense have been widely reported [47,48], but their roles in plant salinity tolerance are poorly understood. The main role of phenolamides in salinity tolerance may be to scavenge ROS [46]. 

**Figure 9:**
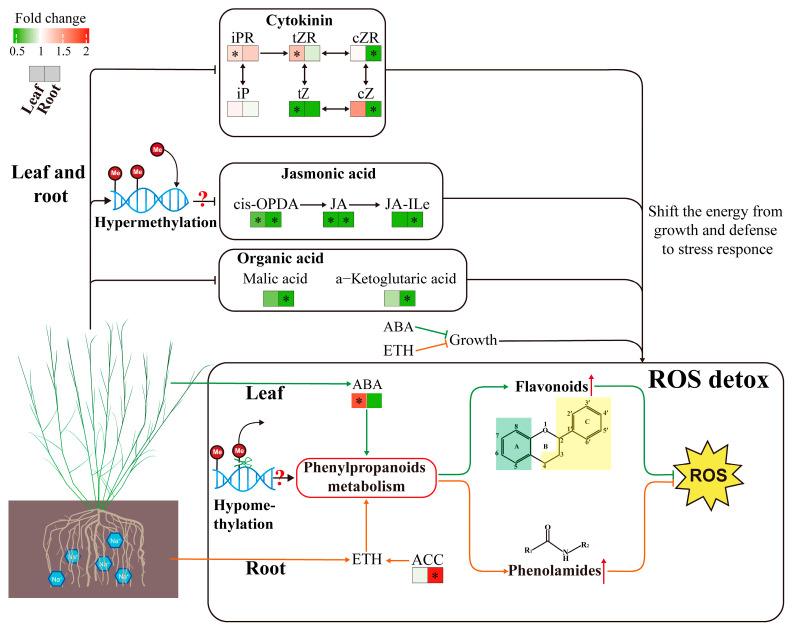
Models of response to long-term salinity stress in *Puccinellia tenuiflora. P. tenuiflora* was irrigated with a nutrient solution containing (stress treatment) or not (control) 300 mM NaCl for two years. The response under long-term salinity stress in the roots was represented by the orange line, whereas the response in the leaves was represented by the green line. The response in both the roots and leaves is represented by the black line. The asterisk indicates a significant difference between and stress treatments (*t*-test, *p* < 0.05). “?” indicates a conjectural role.

Our results indicated that the concentrations of both flavonoids and ABA increased in the leaves but not in the roots, indicating that an increased ABA level may enhance the synthesis of flavonoids in the leaves during the response of *P. tenuiflora* to LSS (Figure 7, Figure 8 and Figure 9). We propose that ABA may mediate significant metabolic changes in the leaves of *P. tenuiflora* under LSS (Figure 9). ABA is the central link in the signal network of the plant salinity stress response [14,15,16]. Under salinity stress, ABA not only regulates plant growth, osmotic regulation, and ion homeostasis but also participates in regulating the balance of ROS [49]. Gai et al. (2020) indicated that the exogenous application of ABA can upregulate the expression of the genes involved in the synthesis of flavonoids, such as anthocyanins, flavonols, and isoflavones, in tea leaves under drought stress [50]. However, in the roots of *P. tenuiflora*, LSS upregulated the concentration of ACC, the precursor of ETH (Figure 8). The previous studies have revealed that ETH plays a positive role in plant salinity tolerance [17,51]. ETH can upregulate the expression of key phenolamide synthesis genes, the shikimate/quinate hydroxycinnamoyl transferase gene, and the putrescine *N*-hydroxycinnamoyl transferase gene [46]. In addition, accumulating evidence suggests that ETH might be a positive regulatory factor for phenolamide synthesis [48]. Overall, during the adaptation of *P. tenuiflora* to LSS, ABA may mediate significant metabolic changes in the leaves, and ETH may mediate significant metabolic changes in the roots to deal with organ-specific ROS stress (Figure 9). Accumulating evidence reveals that CKs play a negative role in plant salinity tolerance, whereas JAs are well known to play core roles in plant defense [52,53,54]. In the present study, LSS decreased the levels of CKs and JAs in both the roots and leaves (Figure 8). The downregulated levels of JAs and CKs in the roots can shift the metabolic substrates and energy from growth and defense against pathogens to the salinity stress response (Figure 9). A similar conclusion was obtained in wheat plants, in which alkali stress shifted the metabolic substrates and energy from general metabolism and defense against pathogens to the stress response through the downregulation of JA, SA, and IAA pathways [55]. Our previous study had shown that in response to LSS, the promoter region of the gene encoding lipoxygenase (a key JA synthesis enzyme) in *P. tenuiflora* underwent hypermethylation, which might lead to a reduction in expression and hence JA concentration [41]. This indicates that JA concentration responded to LSS in *P. tenuiflora* via regulation of DNA methylation (Figure 9).

## 4. Materials and Methods

### 4.1. Plant Growth and Stress Treatment

We collected a single *P. tenuiflora* plant from its natural saline habitat in Changling County, Jilin Province, China, and then transported it to a greenhouse for further propagation and experimentation. Through a process of asexual reproduction according to the method of Li et al. 2021 [41], we generated around 150 ramets from the individual, ensuring that our study utilized a genetically uniform population. The sampling and planting method was described in our previous publication [41]. We selected uniform ramets with 10–11 tillers for our study, which were grown in plastic pots (one ramet per pot) filled with thoroughly washed sand. The size of the pot used was 20 cm in circumference and 12 cm high and contained three holes at the bottom. The ramets were treated with a ½-strength Hoagland′s nutrient solution containing 300 mM NaCl for two years (from March 2018 to March 2020). The control plants were treated with a ½-strength Hoagland′s nutrient solution for two years. Each pot was treated daily with 800 mL of the appropriate treatment solution (Table 1). To avoid the accumulation of NaCl and achieve the desired salt concentration, each pot was thoroughly rinsed once a week with distilled H_2_O (Table 1). The cultivation and stress treatments of experimental materials were conducted in a greenhouse located at Northeast Normal University (43°51′26″ N, 125°19′21″ E). The greenhouse environment was set to maintain temperature at 22–25 °C during the day and 15–18 °C during the night, as well as a 16-h day/8-h night photoperiod. The experimental design used a completely randomized design. We pooled three ramets to represent each biological replicate, with three biological replicates for each of the physiological and biochemical parameters. All mature leaves of each ramet were collected and mixed for all the physiological and biochemical measurements.

### 4.2. Physiological Measurements

To measure photosynthetic parameters, we employed a portable photosynthesis system (LI-6800, Li-Cor, Lincoln, NE, USA). The fully expanded mature leaves of ramets were selected for the measurements of photosynthetic parameters, including the transpiration rate (*E*), net photosynthetic rate (*P_N_*), stomatal conductance (*g_s_*), maximum quantum efficiency of photosystem II (PSII) (Fv/Fm), effective quantum efficiency of PSII (Fv′/Fm′), actual quantum efficiency of PSII (PhiPS2), photochemical quenching (qP), non-photochemical quenching (qN), and electron transfer rate (ETR). We maintained the light intensity at 1200 µmol m^−2^ s^−1^, CO_2_ concentration at 400 mmol mol^−1^, and relative air humidity at 55%. The superoxide anion radical production rate was determined using the method described by Tang (1999) [56]. The fresh tissues were extracted with phosphate-buffered saline (50 mmol/L, pH 7.8). The assay solution contained 0.5 mL enzyme extract and 0.5 mmol/L hydroxylamine hydrochloride, and then the colored reaction was initiated by adding 17 mmol/L sulfanilic acid and 7 mmol/L α-naphthylamine. The concentration of malondialdehyde (MDA) was determined using the method of Tang (1999) [56]. The fresh tissues were homogenized and extracted with 10% TCA. The extract was mixed with 0.6% thiobarbituric acid and heated at 100 °C. The absorbance of the reaction supernatant was measured at 450 nm, 532 nm, and 600 nm. The MDA concentration was calculated according to the formula of Tang (1999) [56]. The electrolyte leakage rate of mature leaves was determined using the method by Tang (1999) [56]. The leaves were collected and washed with deionized water. The leaves were placed in a closed cuvette containing deionized water at 20 °C for 4 h, and then the electrical conductivity (EC_1_) of the solution was measured with a conductivity gauge (DDSJ-308A, Leici Company, Shanghai, China). After this measurement, the cuvette was boiled for 15 min, and then the electrical conductivity (EC_2_) of the boiled solution was measured. The leaf electrolyte leakage rate was calculated as (EC_1_/EC_2_) × 100%.

### 4.3. Widely Targeted Metabolomic Profiling

To detect metabolites in our samples, we used the widely targeted metabolomic method, an innovative metabolomics method that combines the benefits of untargeted metabolomics and targeted metabolomics to achieve high-throughput identification and quantification of plant metabolites associated with plants [57,58]. The freeze-dried samples were first pulverized using a tissue pyrolysis machine (Mixer Mill MM 400, Retsch, Haan, Germany) at a frequency of 30 Hz, and metabolites were then extracted using 1 mL of 70% methanol. A mixture of all the 6 extracts was loaded into a UPLC-MS/MS system (UPLC: Nexera X2, SHIMADZU, Japanese; MS: Applied Biosystems QTRAP, ABSCIEX, Framingham, MA, USA) to construct the MS2 spectral tag (MS2T) library [57,59]. The mobile phase A was 0.1% formic acid, and the mobile phase B was acetonitrile containing 0.1% formic acid. The column temperature was set at 40 °C, and the injection volume was 4 μL. For mass spectrometry parameters, source temperature, ion spray voltage (IS), ion source gas I, gas II, and curtain gas were set at 550 °C, 5500 V (positive ion mode)/‒4500 V (negative ion mode), 50, 60, and 25.0 psi, respectively; the collision-activated dissociation was set at high. The retention time, *m*/*z*, and fragmentation pattern of the detected metabolites were imported into the MWDB-4.0 database (MetWare Biotechnology Ltd., Wuhan, China) to identify the metabolites. The relative concentration of metabolites in each sample was quantified with a multiple reaction monitoring approach according to the workflow of Chen et al. (2013) [57]. 

### 4.4. Quantification of Plant Hormones

Leaf and root hormones were quantified according to the workflow of Shao et al. (2019) [60]. Fresh plant samples were ground into powder in liquid nitrogen, and then plant hormones were extracted with 1 mL of acetonitrile:formic acid:water = 50:1:49, containing stable isotope standards (OlChemIm, Olomouc, Czech Republic). A UHPLC-ESI-MS/MS system (QTRAP 5500 system, AB Sciex, Concord, ON, Canada) was used to detect plant hormones. The parameters of ultra-high-performance liquid chromatography and mass spectrometry used in this study were similar to those described by Lu et al. (2021) [61].

### 4.5. Statistical Analysis of Data

The experimental design was a completely randomized design. The statistical significance of physiological and biochemical measurements was determined by *t*-test (*p*-value ≤ 0.05) with SPSS 16.0 statistical software (IBM, Armonk, NY, USA). Principal Component Analysis (PCA) was carried out using SIMCA 14.1 software (Umetrics, Umeaa, Sweden). Metabolites that differentially accumulated between control and stress treatments were detected using a *t*-test and VIP value in OPLS-DA. The differentially accumulated metabolites were defined by the criteria |Log2 fold change (stress/control)| ≥ 1, VIP > 1, and *p* < 0.05 (*t*-test).

## Figures and Tables

**Table 1:** Treatment method used in this study.

Group	Treatment Solutions	Duration of Treatment	Treatment Methods
CG	Half-strength Hoagland′s nutrient solution without NaCl	2 years	Each pot was treated daily with 800 mL of the appropriate treatment solution. To avoid accumulation of NaCl and achieve the desired salt concentration, each pot was thoroughly rinsed once a week with distilled H_2_O.
SG	300 mM NaCl with half-strength Hoagland′s nutrient solution		

## Data Availability

Data will be made available on reasonable request to the corresponding author.

## Supplementary Materials

The following supporting information can be downloaded at: https://www.mdpi.com/article/10.3390/plants13233445/s1, Table S1: The mean value and standard deviation for Figure 1; Table S2: The mean value and standard deviation for Figure 2; Table S3: The mean value and standard deviation for Figure 3, Figure 4, Figure 5, Figure 6 and Figure 7. Relative concentrations for undetected metabolites were assigned as “9”; Table S4: The mean value and standard deviation for Figure 8.

## Abbreviations

The following abbreviations are used in this manuscript:

LSS	long-term salinity stress
SSS	short-term salinity stress
ACC	1-aminocyclopropane-1-carboxylic acid
PG	phosphatidylglycerol
tZ	*trans*-zeatin
cZ	*cis*-zeatin
iPR	isopentenyladenosine
iP	isopentenyladenine
tZR	*trans*-zeatin riboside
cZR	*cis*-zeatin riboside
